# Economic analysis of uricase production under uncertainty: Contrast of chromatographic purification and aqueous two‐phase extraction (with and without PEG recycle)

**DOI:** 10.1002/btpr.2200

**Published:** 2015-11-28

**Authors:** Mario A. Torres‐Acosta, José M. Aguilar‐Yáñez, Marco Rito‐Palomares, Nigel J. Titchener‐Hooker

**Affiliations:** ^1^ Centro De Biotecnología‐FEMSA, Tecnológico De Monterrey, Campus MonterreyNL64849México; ^2^ Dept. of Biochemical Engineering, The Advanced Centre for Biochemical EngineeringUniversity College London, Torrington PlaceLondonWC1E 7JEU.K.

**Keywords:** uricase, aqueous two‐phase system, economic analysis under uncertainty, Monte Carlo simulation, PEG back‐extraction

## Abstract

Uricase is the enzyme responsible for the breakdown of uric acid, the key molecule leading to gout in humans, into allantoin, but it is absent in humans. It has been produced as a PEGylated pharmaceutical where the purification is performed through three sequential chromatographic columns. More recently an aqueous two‐phase system (ATPS) was reported that could recover Uricase with high yield and purity. Although the use of ATPS can decrease cost and time, it also generates a large amount of waste. The ability, therefore, to recycle key components of ATPS is of interest. Economic modelling is a powerful tool that allows the bioprocess engineer to compare possible outcomes and find areas where further research or optimization might be required without recourse to extensive experiments and time. This research provides an economic analysis using the commercial software BioSolve of the strategies for Uricase production: chromatographic and ATPS, and includes a third bioprocess that uses material recycling. The key parameters that affect the process the most were located via a sensitivity analysis and evaluated with a Monte Carlo analysis. Results show that ATPS is far less expensive than chromatography, but that there is an area where the cost of production of both bioprocesses overlap. Furthermore, recycling does not impact the cost of production. This study serves to provide a framework for the economic analysis of Uricase production using alternative techniques. © 2015 American Institute of Chemical Engineers *Biotechnol. Prog.*, 32:126–133, 2016

## Introduction

Urate oxidase or uricase, (E.C.1.7.3.3) is an enzyme responsible for the breakdown of uric acid into allantoin.^1^ It is a tetrameric protein and each subunit has a mass of 34 kDa.^2^ Because this protein is absent in humans and higher apes due to evolutionary mutations,^3^, ^4^, ^5^ uric acid is the end product of the purine metabolism. This allows the possibility, intensified by a purine‐rich diet, to accumulate uric acid in the bloodstream. When the concentration of uric acid exceeds 7 mg/dL (hyperuricemia), it can precipitate and generate needle‐like crystals,^6^ causing acute pain, inflammation and possible formation of tophi, leading to the development of a type arthritis, known as Gout.^7^

Uricase is an attractive therapy for reducing the uric acid concentration. Allantoin is a soluble molecule in plasma, which can be easily eliminated through the kidneys as it is 5–10 times more soluble than uric acid.^8^ This enzyme has been developed as a pharmaceutical and is expressed in a recombinant host and is delivered as a PEGylated form to improve pharmacokinetics by increasing its size and reducing immunogenicity.^6^, ^9^

Uricase has been isolated and cloned from *Aspergillus flavus*,^10^ *Pseudomonas aeruginosa*,^11^ *Candida utilis*,^12^ etc. Production consists of expression in *E. coli* as an intracellular soluble protein and purification by sequential chromatographic steps.^2^, ^8^, ^13^, ^14^ Recently, recovery by aqueous two‐phase systems (ATPS) has been reported to achieve a high level of recovery and pharmaceutical purity.^15^ This alternative eliminates the use of chromatography in the purification of Uricase. ATPS have been reported to be a less expensive and time consuming operation compared with chromatography, therefore saving resources through process integration and intensification.^16^

One of the main disadvantages of using ATPS, at industrial scale, is the high quantity of materials that need to be disposed of later. Estimates show that the total operation volume can be 10–20 times larger than the input product volume used.^15^, ^17^ With this in mind, research has been conducted to reduce the quantity of materials required by recycling the polymers used, especially polyethylene glycol (PEG).^18^, ^19^, ^20^, ^21^ To accomplish this the basic concept is to use back‐extraction,^18^ which consists of using two sequential ATPS steps, the first recovers the protein in the top phase then on the second ATPS (using the previous top phase as a base) the protein is forced to migrate to the bottom phase. The top phase of the second ATPS can now provide the materials to construct the first ATPS, while the fraction that now contains the protein (bottom phase of the second ATPS) can be filtered to remove the ATPS components and perform a buffer exchange. This could further decrease production costs and also the level of waste disposal.

When designing or improving a bioprocess, it is important to consider possible scenarios that occur during real development. Using bioprocess modelling it is possible to create virtual bioprocesses, allowing input parameters, obtained through research, to be varied so as to obtain estimates of the cost of production per gram (Cost of Goods per gram, CoG/g). Use of such model‐based tools can also help to decrease the costs of research by saving time and resources, reducing the number of experiments and focusing efforts where it is needed.^22^, ^23^ Furthermore, it is possible to incorporate uncertainties that are inherent of any bioprocess.^24^ This area has gained attention recently with publications including those to compare the costs of production between using stainless steel or single‐use equipment,^24^ analysis of pooling strategies in perfusion cultures,^25^ evaluation of batch or continuous cell culture strategies^26^ and the effect of optimization of selected process parameters.^27^ Such research publications provide crucial insights on whether certain strategies are likely to be better than others without the need to carry out extensive experiments.

A range of software tools have been developed to perform bioprocess modelling and economical analysis.^28^, ^29^ These include BioSolve (Biopharm Services, Chesham, Buckinghamshire, UK), SimBioPharma (University College London, London, UK), SuperPro Designer (Intelligen, Scotch Plains, NJ), Aspen Batch Plus (Aspen Technology, Cambridge, MA), etc. BioSolve is an Excel‐based software, that takes into account direct and indirect costs and contains an extensive library for equipment and materials with costs collected directly from suppliers. This software allows the user to create putative bioprocesses and obtain the cost of production, it has a large collection of operations, which can be assembled in many combinations and analyze the impact of each configuration. It also allows to manipulate most of the parameters and obtain results of hypothetical scenarios. The main focus of BioSolve is to obtain the cost of production, while other software is more focused on the engineering part.

This work presents an economic analysis of putative bioprocesses based on reported purification strategies for Uricase. Business related factors were not considered. The article describes the comparison between the chromatographic and ATPS purification techniques by creating bioprocess flowsheets that achieve the same levels of production but which each employ different unit operations. Which parameters affect most the CoG/g were then determined via a sensitivity analysis. A Monte Carlo Analysis was performed on the chromatographic, ATPS and ATPS incorporating a recycling (back‐extraction) step, in each case to mimic real manufacturing behavior. These studies were performed so as to analyze the impact of changing the purification method and later the addition of an unit operation for PEG recycling on the overall process economics.

## Model Set‐Up and Deterministic Analysis

This section details the set‐up of the models in BioSolve used to calculate the CoG/g for the production of Uricase. The models were based on published articles that describe purification with chromatography^2^ and ATPS.^15^ Separately, the ATPS model was modified to include a second ATPS step for the back‐extraction (Uricase movement to salt‐rich phase) and recycling of PEG. The back‐extraction was based on the recovery of a different protein that uses similar conditions for the construction of the ATPS for its recovery.^18^, ^30^ The mechanism of the back‐extraction is explained in detail later in this paper.

**Figure 1:**
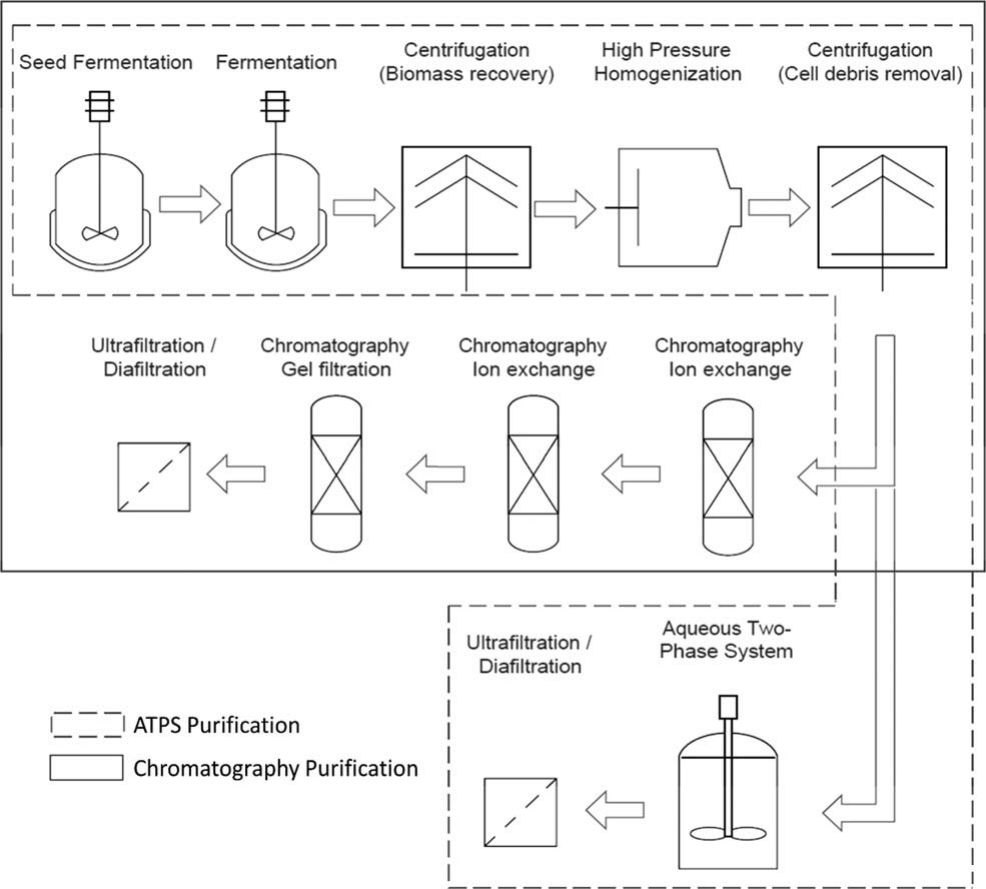
Bioprocesses designed for the production of uricase. Conventional chromatographic process—process using ATPS.

The size of the bioprocesses was based on a chromatographic purification^2^ (the largest production reported for Uricase). This used a 25 L *E. coli* fermentation achieving a titer of ∼0.5 g/L. The chromatographic process was modified slightly by the addition of a centrifuge for biomass recovery, high pressure homogenization to release the intracellular material and a second centrifuge for cell debris removal. An additional unit operation at the end of the process was added; an Ultrafiltration/Diafiltration (UF/DF) step for concentration and buffer exchange. For the ATPS bioprocess, the three chromatographic columns were changed to a single ATPS step. The UF/DF was used to remove ATPS components while concentrating the sample. The sequences of operations are shown in Figure 1. Recovery yields were taken from their respective publications,^2^, ^15^ for the chromatographic process the final yield is 43.2% and for ATPS process is 66%. The added unit operations (centrifuges and homogenization) were used according to the default parameters provided by BioSolve.

A relevant comparison can only be made if both processes achieve similar purity. The ATPS operation is based on the production from *Candida utilis*, but analysing both bioprocesses in their respective publications,^2^, ^15^ it is found that they can achieve a specific activity (enzyme units per mg of total protein) of 27 U/mg. In terms of proteins, it means that they have the same purity, but in a real crude extract there are other contaminants (for example nucleic acids), the ATPS report doesn't include information on this, but as the product will have a pharmaceutical use, additional operations need to be included in both processes (viral inactivation, nucleic acids removal, etc.), but as these are polishing operations they were excluded from this analysis to focus on contrasting chromatography and ATPS.

It is important to mention that both bioprocesses achieve 27 U/mg, but the chromatographic process achieves 25.7 U/mg in the first two chromatographic columns, the third improves this to 27 U/mg, the complete chromatographic process was considered for the analysis as this can achieve a purity of 99% and a 3 sequential array of chromatographic columns is the most reported form to purify Uricase.

Both bioprocesses were assumed to achieve the same titer, require identical reactor volumes, operators (Labor) and Quality Control (QC) tests. Labor was set to be 13% of the total CoG/g, as it has been reported to be between 10 and 15% for recombinant protein production^23^ with the exact wage values set to the average for the United Kingdom.^31^ QC test were modified from the default in BioSolve, as they are focused to monoclonal antibodies (Mabs), most of them were preserved but those specific to Mabs were excluded, in this case only the measurement of residual protein A was left out. The costs of equipment and material needed for the processes were obtained from the library included in the BioSolve software. The only other inputs were the media and solutions used, particularly for the fermentation, ATPS solutions and chromatographic buffers.

**Figure 2:**
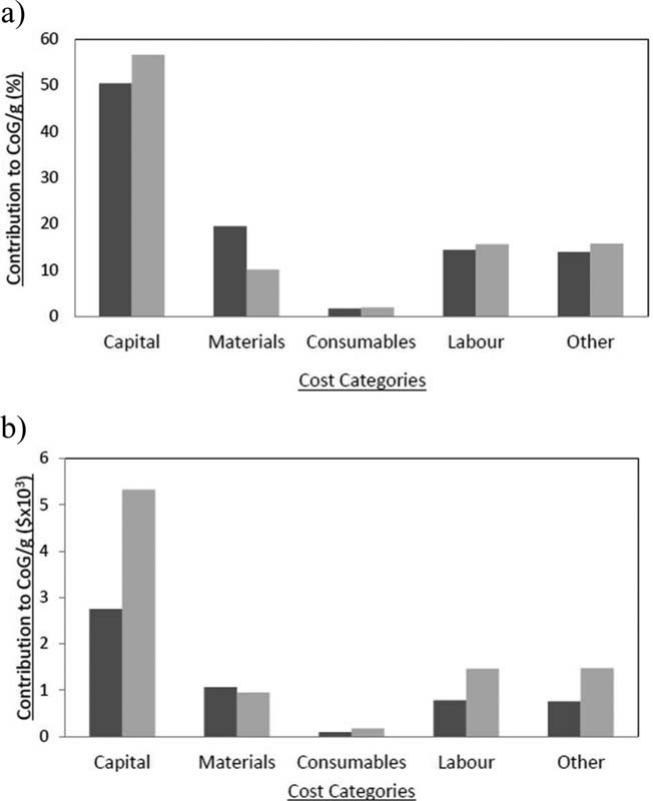
Breakdown per cost categories of the CoG/g obtained in the deterministic analysis. (a) Shows the breakdown according to percentages, while (b) shows the breakdown in absolute terms. 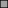
Chromatographic‐based process; 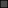
ATPS.

After setting‐up the models, the CoG/g for the base scenario were returned as ∼$9,400 and ∼$5,450 for the chromatographic and ATPS processes, respectively. The ability of BioSolve to break down the CoG/g to its components was used to identify the main contributors. In Figure 2, it can be seen that the costs for capital, consumables, labor, and others are lower for the ATPS bioprocess, while materials were lower for the chromatographic purification. This reflects the nature of each bioprocess. Chromatography has larger capital costs because of the need to acquire more equipment than ATPS process. ATPS recovery needs the input of large quantities of material to construct each system while chromatography requires new resins with constant turnover and a larger level of consumption of consumables. The difference in consumables usage between the two process options is not as large as for materials due to the fact that the ATPS bioprocess requires a larger supply of UF/DF filters because of the nature of the operation which requires significant volumes to be filtered in order to remove the PEG from solution, each filter has a certain limit of volume to process and by filtering more the lifetime becomes shorter.

## Sensitivity Analysis

The values of the parameters that determine the CoG/g in a bioprocess are not constant. Parameters that affect the CoG/g the most in a bioprocess can be fermentation titer, desired final production (target output), downstream processing yield (DSP yield), material costs and labour wages.^24^, ^25^, ^32^ To capture the essence of this, a sensitivity analysis was performed in which a best and worst case scenario were created for every parameter. Target output was held constant so as to provide a common basis for the comparison between the process options. The sensitivity analysis was carried out by changing systematically each parameter individually. Table 1 shows the scenarios used in this sensitivity analysis.

**Table 1:** Scenarios Used for the Sensitivity Analysis

	Scenarios		
Variable	Best	Base	Worst
Titer (g/L)	0.577	0.484	0.392
DSP Yield (±%)	+10	0	−10
Material Cost (±%)	−25	0	+25
Labor (Location)	Mexico	UK	USA
Production operator ($)	41,872	32,935	37,689
Production supervisor ($)	52,337	38,490	37,689
Quality assurance ($)	70,149	56,176	35,995
QC ($)	39,101	31,400	35,995

The titer range was developed by obtaining the mean (±1 standard deviation) from the calculated titer needed to yield the level of production cited for a process based upon chromatographic purification.^2^ According to the literature,^24^, ^25^, ^32^ DSP yield was modified by ± 10% and material costs by ±25%, for those materials that affect the most; fermentation media (2× YT medium), ATPS materials (PEG2000, ammonium sulfate, sodium chloride) and UF/DF filters. Labor wages were adjusted to the prevailing pay rate in two countries selected to have higher and lower salaries compared to the United Kingdom, respectively. The United States (US) was chosen as the former because of the large concentration of biotechnology companies and suppliers and Mexico for the latter as it offers comparatively lower wages.

**Figure 3:**
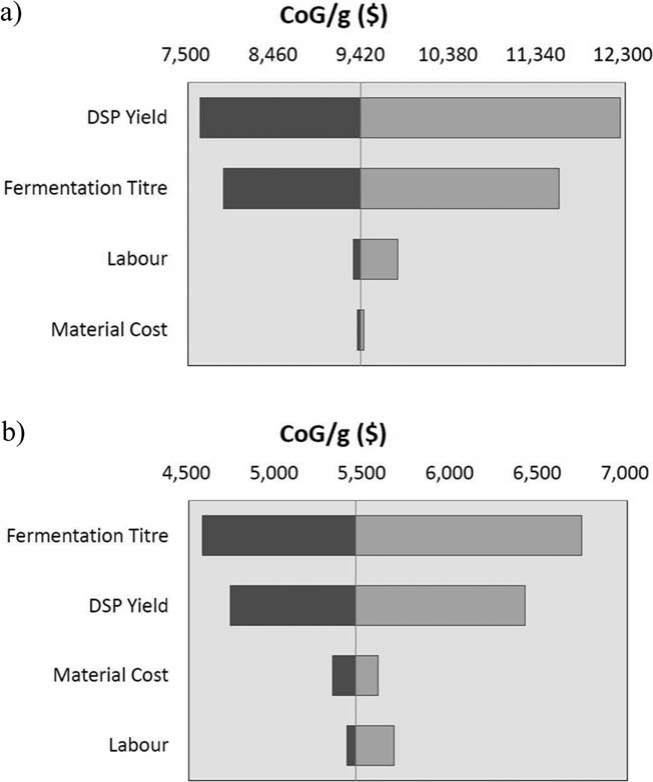
Tornado plot for the results of the sensitivity analysis. (a) Chromatographic‐based process (vertical axis cross at base scenario: $9,396.97) and (b) ATPS‐based process (base scenario: $5452.00).

Figure 3 shows the results of the sensitivity analysis. In both bioprocesses, the two key parameters were fermentation titer and DSP yield. In the case of chromatographic purification, DSP yield was the most important parameter. A slight change had a larger impact because of the extended process sequence, while in the ATPS case, with fewer unit operations, DSP yield had less impact and the fermentation titer dictates the final production and therefore had the bigger impact on the CoG/g. The third and fourth parameters ranked in importance were labour and material costs. In the ATPS process, materials were a bigger concern as they are required in large quantities to construct the system, but there is also a need for more UF/DF filters to remove the PEG from the solution. On the other hand, chromatographic resins can be reused and in bind‐and‐elute processes samples can be concentrated whereas in ATPS they are diluted. This feature means that materials costs have a lower impact on the chromatographic‐based purification process.

At this point in the analysis, the impact of changes in individual parameters had been analyzed. Moreover in a real process, parameter changes occur simultaneously and it is important to analyze them in a synergistic fashion as described next.

## Monte Carlo Analysis

**Figure 4:**
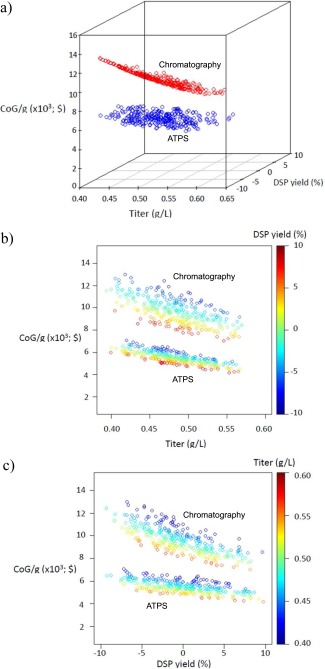
Monte Carlo Analysis results examining the impact of titer and DSP yield on CoG/g for Chromatographic‐based and ATPS without recycle processes. (a) 3‐D representation of both CoG/g distributions. (b) 2‐D representation of distributions with titer in the x‐axis and color variable for DSP yield, while (c) has the DSP yield on the x‐axis and titer as color variable.

A Monte Carlo analysis was performed to understand how CoG/g behaves when key parameters are allowed to vary simultaneously. The top two parameters from the sensitivity analysis were examined (Fermentation titer and DSP yield). To perform this analysis a program was written in Visual Basic to generate random values for both parameters as inputs to BioSolve, which in turn computed the corresponding CoG/g values. A probabilistic function was used to generate random values for the parameters. A triangular distribution with the same limits (most‐common, maximum and minimum) as in the sensitivity analysis was assumed as this is a frequently used distribution in the bioprocesses literature.^26^, ^33^ A moving average (MA) was calculated after each run with stable outcomes achieved after ∼300 simulations. This was taken as a standard for the Monte Carlo analysis. Figures 4a–c shows the results for the simulation runs.

**Figure** 4 **a** shows the complete results in a tridimensional environment. Simplified views are provided in Figures 4b,c. Having either a higher titer or a DSP yield results in a lower CoG/g, while a decrease in titer and DSP yield produces a higher CoG/g. If high titer and high DSP yield are combined the lowest possible CoG/g is achieved. Analysing Figures 4b,c, it can be seen that as the CoG/g decreases it also tends to stabilize. It can be inferred that there is a condition for titer and DSP yield where they are no longer the main contributors to the change of the CoG/g. This means that there is a critical point where the CoG/g is dependent on a product‐oriented parameter rather than on a process‐oriented parameter. It is reported elsewhere,^27^ when titer (a process parameter) was optimized the main parameter became target‐output (a product‐parameter). A zone exists where both chromatographic and ATPS purification based processes overlap and have a similar CoG/g. This occurs when the chromatographic‐based bioprocess is operated at best conditions (the highest titer and DSP yield) and ATPS recovery operated at worst conditions.

**Figure 5:**
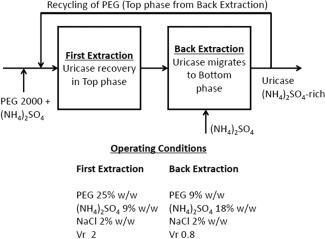
Representation of the back‐extraction used in the third, putative, bioprocess. The first system is constructed by the mixing of the sample (coming from the centrifuge after cell debris removal), PEG and ammonium sulfate, the bottom phase is discarded. The second system uses the top‐phase from the previous system as a source of material but still has a fresh input of (NH_4_)_2_SO_4_, the top‐phase is now returned as material for construction of the first system. The bottom‐phase enriched in Uricase is used for further purification.

**Figure 6:**
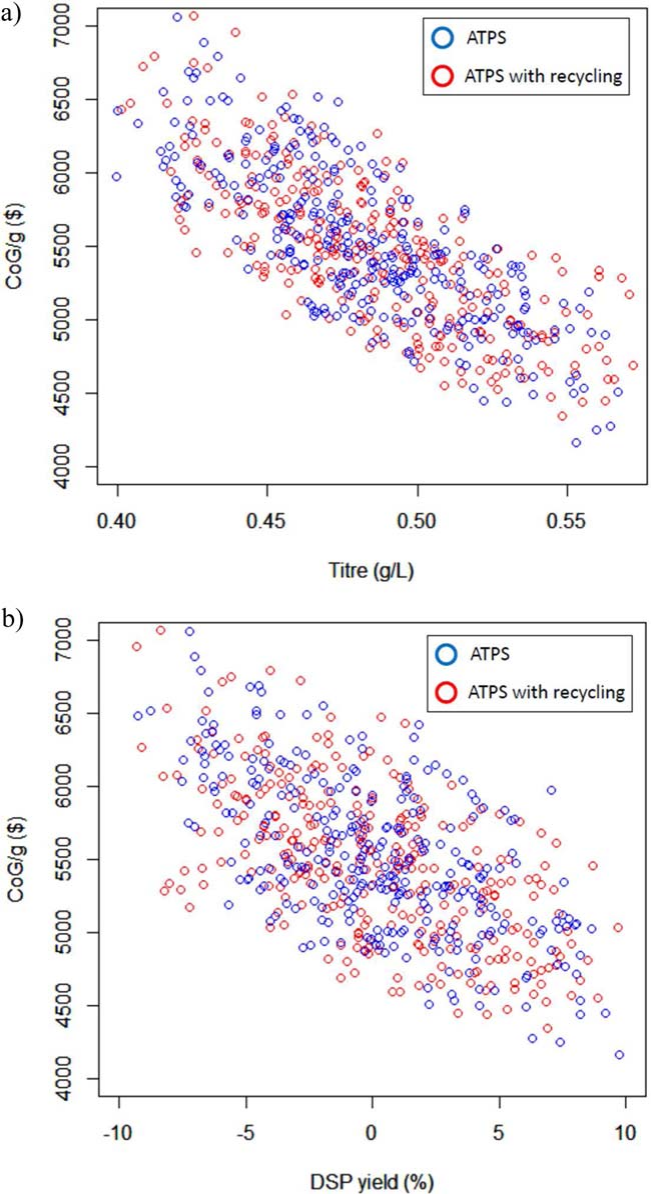
Comparison of the results of Monte Carlo analysis for ATPS with and without recycle. (a) Impact of titer and (b) impact of DSP yield.

An additional bioprocess based on recycling the ATPS was included. In this, a second ATPS system is added where Uricase migrates to the bottom phase leaving a PEG‐rich top phase that can be reused as material for the first ATPS. According to the literature,^17^, ^18^ this can be achieved when the percentage of salt (in this case ammonium sulfate) is greater than that of the polymer and the volume of the bottom phase is greater than the top phase volume. Figure 5 shows how the components of the systems move between both elements of the ATPS and the conditions necessary for each extraction. Recycling with back‐extraction allows reuse of 60% of the PEG, 20% of (NH_4_)_2_SO_4_ and 20% of NaCl.^18^ This mode of operation reduces the cost of the first ATPS by reducing the total inventory of materials needed. The process based upon recycling and back‐extraction was compared with the traditional ATPS and the results are shown in Figure 6. It can be seen there is not a clear difference between the two options. The deterministic result for the new bioprocess CoG/g is $5430, slightly lower than the simple ATPS bioprocess. When Monte Carlo analysis was applied the distribution of costs was almost identical.

Even though the addition of recycling decreases significantly the amount of material required for every batch, the CoG/g does not decrease at the same rate because of the dominance of the UF/DF operation. In the modified process there is a significant level of dilution. The second ATPS was made taking into account the solute composition passed on from the previous top phase. The volume input to the second ATPS is only a fraction of the total volume. The output collected for further purification was 56% of the second system, which equates to a final dilution of 10 times the initial sample input and hence a large volume increase but a lower PEG concentration compared to the original top phase from the ATPS bioprocess without recycle. UF/DF is still needed to remove the salts and residual PEG. This causes an increase in the cost of the UF/DF operation, because more volume is being filtered. Although recycling decreases the costs of making up the ATPS this saving is not sufficient to decrease the final overall CoG/g appreciably.

Linear models were generated for each of the 3 comparative processes and the results are presented on Table 2. All of the parameters and the intercepts are significant at a value of *α* = 0.01. The values for the coefficients are consistent with the graph in Figure 4a. When titer and DSP yield change, they impact most the CoG/g for the chromatography bioprocess, while the CoG/g changes more slowly for the ATPS bioprocesses (both with and without recycle). Linear models also showed negligible CoG/g differences between ATPS with and without recycle. The DSP yield coefficient was slightly greater in recycling, but this might be an artefact of the addition of an unit operation. The same situation was presented during the sensitivity analysis, where the chromatographic‐based bioprocess was seen to be more sensitive to changes in DSP yield due to the larger number of process steps compared withATPS.

**Table 2:** Linear Models Calculated for CoG/g in Terms of Titer and Downstream Processing Yield (*P*‐values × 10^−16^).

	Chromatography		ATPS		ATPS with Recycle	
	Coefficient	*P*‐value	Coefficient	*P*‐value	Coefficient	*P*‐value
Intercept	191,956	<2	11,013	<2	10,917	<2
Titer	−19,883	<2	−11,374	<2	−11,224	<2
DSP Yield	−227	<2	−83	<2	−83	<2

Analysis of the linear models revealed, as mentioned before, an area of overlapping CoG/g as follows: chromatographic‐based process requires to operate in the top 23% of all possible values obtained under the probabilistic function, while the ATPS needs to operate in the bottom 52%. These results are not promising as the chromatographic purification has been extensively optimized already. For the ATPS option, there is still space for optimization, which offers scope to reduce the overlapping area by optimizing the ATPS recovery and therefore decreasing the CoG/g through the use of this unit operation in preference to chromatography. The result is indicative of the potential for ATPS to be used in commercial bioprocesses.

There are other potential strategies to reduce the amount of waste generated by the ATPS processes and to recycle material. When using back‐extraction, to achieve a significant decrease in the CoG/g, it is important to decrease the volume to be filtered and hence reduce the dominance of the UF/DF. In the back‐extraction employed in this study, the bottom phase volume was larger than the top phase. It is however possible to tailor a ATPS for this particular protein such that the relative volume < 1 (ratio of volumes of the top and bottom phases) whilst still maintaining a high level of recovery and purity. This should also ensure that the volume to be processed by the UF/DF remains comparable to that of the simple ATPS process studied here. One potential alternative strategy is to saturate the phase to which the protein of interest migrates. This requires the system to be set‐up as before but then to remove the phase which is rich in contaminants; for this system the bottom phase. After that fresh components are added to the preserved top phase to form a new bottom phase and a new sample added. Uricase will once more migrate to the top phase, but now as this phase is already loaded with protein the efficiency of capture will be reduced. Experimental research can locate the balance between the number of cycles for saturation and protein recovery, whilst still achieving a decrease in the CoG/g.

## Conclusions

This article describes an economic analysis of three possible routes for the production of Uricase for pharmaceutical use carried out using a commercial software package. It was possible to identify the key parameters that affect most the costs of production for each bioprocess studied: conventional chromatographic‐based purification, simple ATPS use and ATPS with phase recycle. A series of Monte Carlo simulations were performed to understand how much the distribution of CoG/g behaves when uncertainty in process parameter values was considered. It was found that when titer and DSP increase CoG/g decreases but toward a stable outcome, suggesting an increase of the level of importance of product‐oriented variables. According to the linear models fitted to the dataset, an area of overlap exists where the ATPS and chromatographic‐based purification processes have similar CoG/g.

A third putative bioprocess was incorporated based on ATPS but which included recycling of materials. This option decreased the amount of materials needed but the CoG/g did not decrease significantly due to an increase of the unit operation costs which reflect the high volumes of material which have to be processed in such a process option. Methods to achieve a lower CoG/g for ATPS with recycling are briefly discussed. Performance is strongly dependent upon the ability to optimize phase recycling.
